# Classification of Traditional Chinese Medicine Syndromes in Patients with Chronic Hepatitis B by SELDI-Based ProteinChip Analysis

**DOI:** 10.1155/2012/626320

**Published:** 2012-05-30

**Authors:** Ya-Nan Song, Hui Zhang, Yan Guan, Jing-Hua Peng, Yi-Yu Lu, Yi-Yang Hu, Shi-Bing Su

**Affiliations:** ^1^Research Center for Traditional Chinese Medicine Complexity System, Shanghai University of Traditional Chinese Medicine,1200 Cailun Road, Pudong, Shanghai 201203, China; ^2^Institute of Liver Diseases, Shuguang Hospital, Key Laboratory of Liver and Kidney Diseases of Ministry of Education, Shanghai University of Traditional Chinese Medicine, Shanghai 201203, China

## Abstract

Traditional Chinese medicine (TCM) syndrome, also called ZHENG, is the basis concept of TCM theory. It plays an important role in TCM practice. There are excess and deficiency syndromes in TCM syndrome. They are the common syndromes in chronic hepatitis B (CHB) patients. Here we aim to explore serum protein profiles and potential biomarkers for classification of TCM syndromes in CHB patients. 24 healthy controls and two cohorts of CHB patients of excess syndrome (*n* = 25) or deficiency syndrome (*n* = 19) were involved in this study. Protein profiles were obtained by surface-enhanced laser desorption ionization time-flight mass spectrometry (SELDI-TOF/MS) and multiple analyses were performed. Based on SELDI ProteinChip data, healthy controls and CHB patients or excess and deficiency syndromes in CHB patients were obviously differentiated by orthogonal partial least square (OPLS) analysis. Two significant serum proteins (m/z 4187 and m/z 5032) for classifying excess and deficiency syndromes were found. Moreover, the area under the receiver operating characteristic (ROC) curve was 0.887 for classifying excess and nonexcess syndrome, and 0.700 for classifying deficiency and nondeficiency syndrome, respectively. Therefore, the present study provided the possibility of TCM syndrome classification in CHB patients using a universally acceptable scientific approach.

## 1. Introduction

Traditional Chinese medicine (TCM) syndrome classification (also defined as Zheng differentiation) and treatment is the basis concept of TCM theory. TCM syndrome, a profile of symptoms and signs as a series of clinical phenotypes, plays an important role in understanding the human homeostasis and guiding the applications of Chinese herbs and acupuncture. Heat, cold, excess, and deficiency are the four basic syndromes of maladjustment nature in TCM [1]. Damp heat stasis syndrome and liver and kidney Yin deficiency syndrome, classified as excess syndrome and deficiency syndrome, respectively, are the common syndromes in chronic hepatitis B (CHB) patients [2]. Excess syndrome refers to the accumulation or stagnation of metabolic waste, body fluids, and blood, whereas deficiency syndrome means to “overcatabolism” and “overconsumption”, the deficiency of nutrients, and weakness [1].

So far, an experiential diagnosis approach has been always used to classify excess syndrome and deficiency syndrome in CHB patients. TCM practitioners with rich experience in TCM diagnosis and treatment are often able to improve the symptoms of CHB patients, which may be considered to be untreatable by conventional medicine [3]. Lu et al. [4] mentioned that for coronary heart patients with different TCM syndromes, if herbal medicine was appropriate to TCM syndrome, the effective rate would increase. It was suggested that syndrome classification acts as a pivot in the therapeutic process and directly affects the therapeutic result of a specific disease. Instead of experiential diagnosis, therefore, it is necessary to standardize the diagnosis criteria for classification of excess and deficiency syndromes in patients with CHB by using a universally acceptable scientific approach.

Proteomics, a rapidly evolving tool in systems biology of analyzing protein expression in a comprehensive degree, is widely applied for disease diagnosis and prognosis, such as brain injury [5], appendicitis [6], liver fibrosis [7], and esophageal cancer [8]. Surface-enhanced laser desorption ionization time-flight mass spectrometry (SELDI-TOF/MS), a powerful tool for global analysis of protein expression, provides an efficient and sensitive method for biomarker discovery. It can obtain the spectra composed of hundreds of protein peaks, each characterized by its mass-to-charge ratio (m/z) and each area represented by its amount [9]. Considering the features of measuring in a high-throughput way and analyzing with a small amount of materials, SELDI-TOF/MS has become an attractive tool for clinical application. The technology has successfully led to the discovery of new biomarkers for diagnosis and treatment of various diseases, for example, accurate diagnosis of early hepatocellular carcinoma [10] and laryngeal carcinoma [11], and identification of treatment efficacy-related host factors in chronic hepatitis C [12].

CHB is a kind of global infective disease induced by hepatitis virus B (HBV). It is estimated that about 400 million people are suffering from HBV infection worldwide [13, 14]. And HBV leads to 500,000 to 1.2 million deaths every year because of turning into liver cirrhosis and hepatocellular carcinoma (HCC) [15]. With 120 million people infected with HBV, China has the largest population in the world. And among them, about 30 million people are suffering from CHB [3]. TCM is widely used in the treatment of CHB and was found to be effective in China [16–18], and conventional medicine hardly heals CHB patients completely, so more and more people therefore turn to get help from TCM. In the present study, we aim to use SELDI-TOF/MS analysis and related data processing methods to find the protein profiles of excess and deficiency syndromes and the promising protein biomarkers to classify these TCM syndromes in patients with CHB.

## 2. Material and Methods

### 2.1. Study Population

The study has been approved by Shuguang Hospital, the affiliated hospital of Shanghai University of TCM. Serum samples were collected from November 2009 to July 2010. The experiment involved 24 healthy controls and two cohorts of CHB patients of excess syndrome (*n* = 25) or deficiency syndrome (*n* = 19). The demographic and clinicopathological data about the participants were showed in Table 1. The differences of gender and age have no statistical significance among three groups (*P* > 0.05). The selected 44 patients with CHB must be in accordance with the following criteria: (1) all patients were diagnosed according to both CHB and TCM syndromes and confirmed by chief physicians; (2) the diagnosis of CHB was based on the guideline defined by the Chinese Society of Hepatology and Chinese Society of Infectious Diseases in 2005 [19]; (3) the TCM syndrome differentiation was referred to the viral hepatitis diagnostic standard described by the Internal Medicine Hepatopathy Committee of Chinese Traditional Medicine Association in December, 1991 [20]. An informed consent was signed by each of the participants, and the study protocol conformed to the ethical guidelines of the Declaration of Helsinki (1964).

The fasting blood samples were collected from two experimental groups of patients with CHB and healthy controls in the morning and allowed to stand for 30 min at room temperature and then centrifuged at 1,5000 rpm for 10 min. All the serum samples were stored at −80°C until further analysis.

### 2.2. Protein Profiling by SELDI-TOF/MS

CM10 (Ciphergen Biosystems, Fremont, CA, USA) was used to further serum differential protein spectrum analysis. First, 5 *μ*L of the cleared serum was mixed with 10 *μ*L of U9 solution containing 9 mol/L urea, 2% CHAPS, 50 mmol/L Tris/HCl, and 1% DTT (pH 9.0; Sigma, USA). Subsequently, the previous sample was diluted with 185 *μ*L CM10-binding buffer (50 mmol/L sodium acetate, pH 4.0; Sigma, USA) to give a final dilution of 40-fold. In addition, the array spots should be preactivated twice with 200 *μ*L of binding buffer for 5 min. And then, 100 *μ*L of diluted serum samples was loaded on each array spot and incubated with shaking for 1 h at 4°C. Two washes with binding buffer and one quick rinse with HPLC grade water were continued to remove nonselectively bound proteins. After air-drying, 0.5 *μ*L of freshly prepared sinapinic acid solution in 0.5% trifluoroacetic acid and 50% acetonitrile was added on each spot for twice. The chips were ready for MS detection when dried.

 Mass accuracy was calibrated externally by using the all-in-one peptide molecular mass standard. After calibration passed, the chips were scanned by SELDI-TOF/MS in a PBS-Iic ProteinChip reader (Ciphergen Biosystems) to measure the masses and intensities of the protein peaks. According to experience, many parameters were optimized for getting more protein peaks and separating these peaks better. At last, the reader was set up as follows: laser intensity, 100; laser sensitivity, 8; optimized mass range, 2,000–15,000 Da; focus mass, 8,500 Da; high mass, 50,000 Da; and data acquisition parameters, 25 delta to 5 transients per to 10 ending position to 75. Data were processed automatically using the Ciphergen Protein-Chip Software (version 3.1.1, Ciphergen Biosystems). Spectra were normalized, calibrated, and aligned.

### 2.3. Data Processing

Protein spectra were automatically generated after all raw data were collected. The profiling spectra of serum samples were first normalized using total ion current by Ciphergen ProteinChip Software 3.1.1. Peak selection was carried out by the Biomarker Wizard program. Protein peaks were selected based on a first pass of signal-to-noise ratio of 5. This process was completed with a second pass of signal-to-noise ratio of 2, and peak selection at 0.3% of the mass window, and the estimated peaks were added. After the preliminary analysis of protein spectra, these selected protein peaks were exported to other commercially available software for further analysis.

 The statistical analysis was performed by SPSS software (version 15.0, Chicago, IL, USA). Values are expressed as the mean ± SD. The baseline characteristics were compared using appropriate method. For continuous variables, one-way factorial analysis was used, or the Wilcoxon rank-sum test was used because of the skewed distributions. And for categorical variables, x^2^ test was used. Multivariate analysis was carried out to determine the independent variables associated with differentiation of syndromes. Two-sided *P* value <0.05 for one-way factorial analysis or adjusted *P* value <0.0167 for Wilcoxon rank-sum test was considered statistically significant. SELDI-TOF/MS-measured variables showing statistical significance on univariate analysis were subjected to binary logistic regression to determine significant independent factors. After the regression, the values of the prediction probability were applied to the classification of the samples. Then receiver operating characteristic curve (ROC) was made by using the SPSS software.

 The preprocessed data obtained by Ciphergen ProteinChip Software were also exported and analyzed by principle component analysis (PCA) and orthogonal partial least squares (OPLSs) using the SIMCA-P software (version 11.5, Umetrics AB, Umea, Sweden).

## 3. Results

### 3.1. Clinical Characteristics of Study Population

Clinical characteristics and TCM syndromes in CHB patients and healthy controls are shown in Table 1. Data including body mass index (BMI), alanine aminotransferase (ALT), aspartate aminotransferase (AST), *γ*-Glutamyltransferase (GGT), alkaline phosphatase (ALP), albumin (ALB), triglyceride (TG), bile acid (BA), total bilirubin (TBIL), prothrombin time (PT), Hepatitis B surface antigen (HbsAg), and HBV DNA were expressed as the mean ± SD. According to the statistical analysis, no clinical factors were significantly different between excess syndrome and deficiency syndrome, indicating that the two TCM syndromes could not been classified by the general clinical parameters of CHB.

### 3.2. Serum Protein Profiling by SELDI-TOF/MS

**Figure 1:**
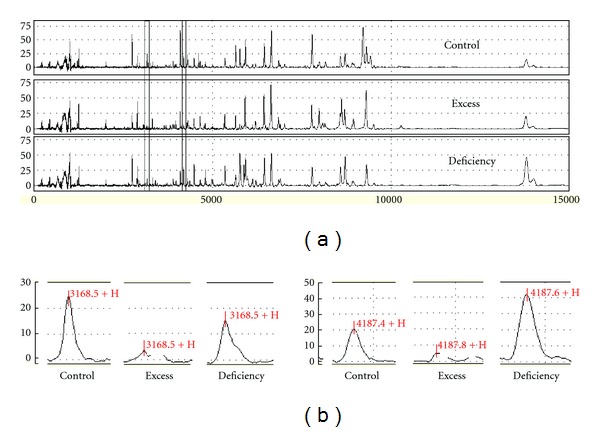
Representative protein profiles of serum samples of healthy controls and patients with CHB of excess symptom and deficiency syndrome. Protein peak spectrum of serum was analyzed by the SELDI-TOF/MS system, and representative protein peaks within m/z 0–1,5000 of three groups are shown (a). Statistically significantly different peaks between excess syndrome and deficiency syndrome are shown in the enlarged view, m/z 3168 on the left and m/z 4187 on the right (b).

Using the SELDI ProteinChip system, we analyzed the serum protein profiling from 24 healthy controls, 25 excess syndrome patients with CHB, and 19 deficiency syndrome patients with CHB. Peaks were detected automatically after baseline subtraction. 184 protein peaks were detected and these peaks were overlapping among 3 groups.  Figure 1(a) displays the representative protein profiling obtained by SELDI-TOF/MS analysis showing the protein peaks of healthy controls and CHB patients of two different TCM syndromes. As shown, the SELDI technology was effective in separating low molecular weight proteins and polypeptides between m/z 2,000 and m/z 15,000.

### 3.3. Classification of TCM Syndromes by Pattern Recognition Analysis

**Figure 2:**
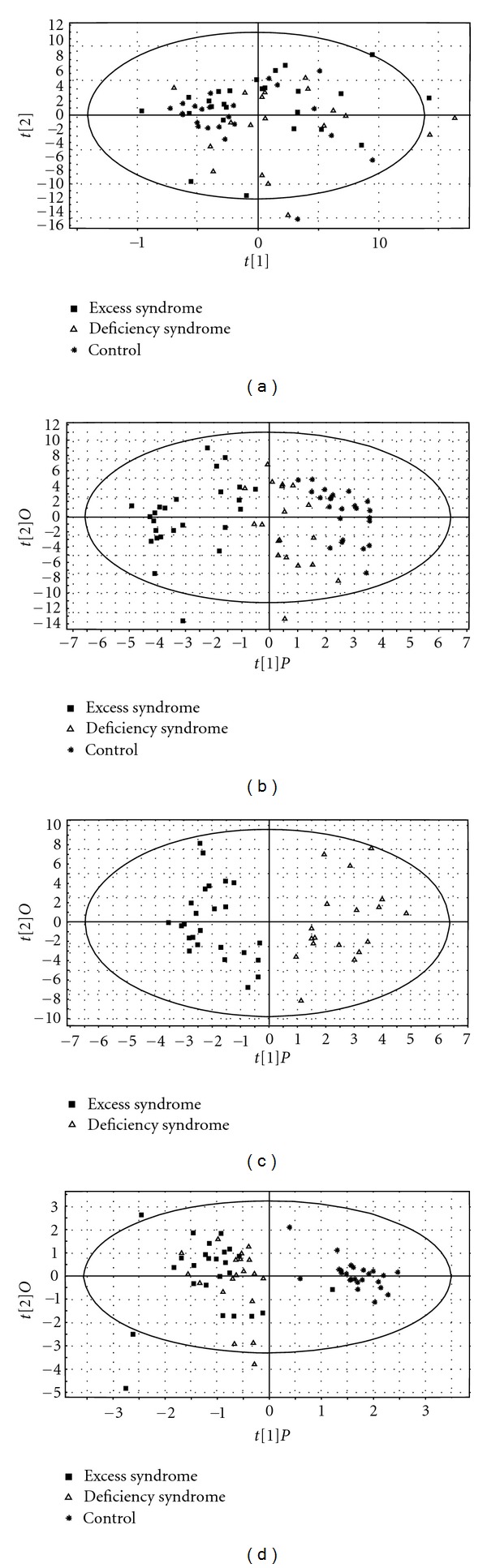
PCA score plot and OPLS score plots of 25 CHB patients of excess syndrome (▪), 19 CHB patients of deficiency syndrome (∆), and 24 healthy controls (∗) based on the serum protein profiling detected from SELDI-TOF/MS or the clinicopathological data of each individuals. (a) PCA score plot among the control group and CHB groups of excess syndrome and deficiency syndrome; OPLS score plots (b) among the control group and CHB groups of excess syndrome and deficiency syndrome and (c) between excess syndrome group and deficiency syndrome group. (a)–(c) Models of score plots were constructed by the data from SELDI-TOF/MS. (d) Another OPLS score plot among the three groups using clinical parameters.

To explore whether the serum protein profiles could help to classify excess syndrome and deficiency syndrome in CHB patients, pattern recognition analysis was carried out to analyze the data generated by SELDI-TOF/MS. Principle component analysis (PCA) was first used as an unsupervised statistical method to study the protein differences among the three groups. The result showed that there was not a trend of separation between control group and CHB group or excess syndrome and deficiency syndrome groups (Figure 2(a)). Then a supervised statistical method, that is orthogonal partial least squares (OPLSs) analysis, was performed as mentioned before. As OPLS score plots were displayed, a tendency of separation was observed among the three groups (Figure 2(b)), and an obvious separation exists between excess syndrome group and deficiency syndrome group (Figure 2(c)), indicating that the whole protein expression was different not only between healthy controls and CHB patients but also between excess and deficiency syndromes in CHB patients.

On the other hand, to investigate whether clinical parameters had influence on classification, the PCA model comparing three groups was constructed using clinicopathological data alone. But the result was not satisfying and the groups could not be differentiated from each other (not shown). And then the OPLS model was carried out. As shown in Figure 2(d), only the control group could be separated from the two others, whereas the TCM syndrome groups could not be separated from each other. It was suggested that the general clinical data were good at classifying health and HBC, while the data from SELDI-TOF/MS could be used for TCM syndrome classification. 

### 3.4. Serum Protein Potential Biomarkers of TCM Syndromes

**Figure 3:**
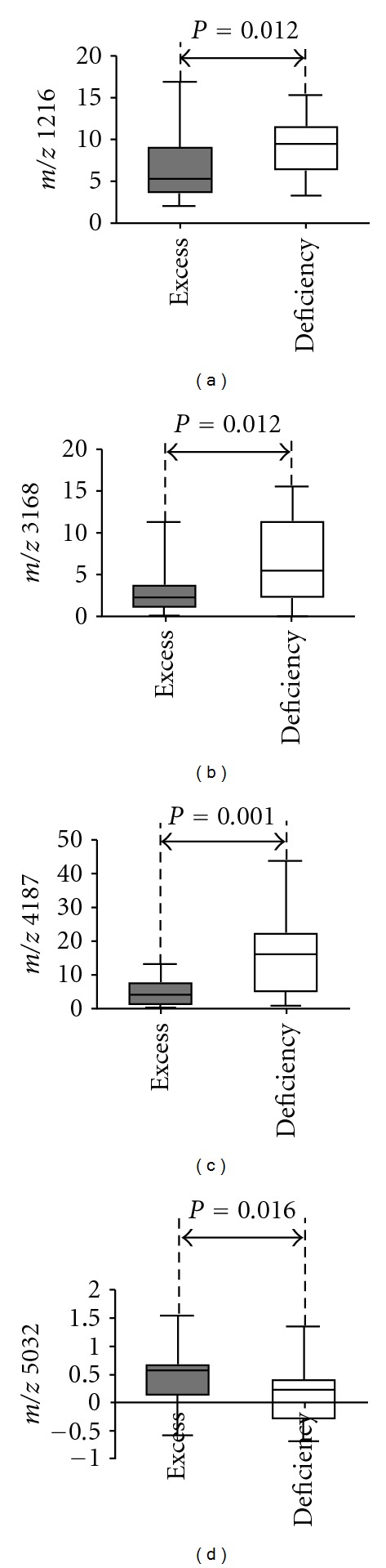
Box-plots for protein peak comparison between TCM syndrome groups. Proteins m/z 1216 (a), m/z 3168 (b), and m/z 4187 (c) were in lower abundance in excess syndrome group than those in deficiency syndrome one, while protein m/z 5032 (d) was in higher abundance.

Among a total of 184 protein peaks detected, 4 significantly different peaks were observed between excess and deficiency syndromes according to Wilcoxon rank-sum test. Three of four protein peaks were in lower abundance in excess syndrome group (Figures 3(a), 3(b), and 3(c)), and the remaining one was in higher abundance (Figure 3(d)). These statistically significant differences can be displayed clearly in the box-plots. Also, an enlarged view of m/z 3168 and m/z 4187 is shown in Figure 1(b). So they may be potential biomarkers for classifying excess syndrome and deficiency syndrome with CHB.

### 3.5. Logistic Regression Analysis

**Figure 4:**
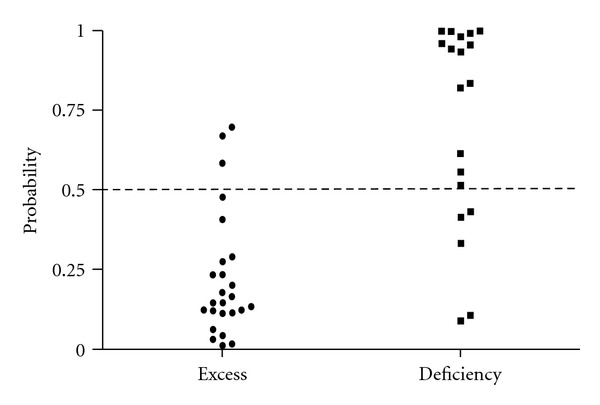
Diagnostic potential of the two marker proteins (m/z 4187 and m/z 5032) using binary logistic regression method with the data from different TCM syndromes in CHB patients. 88% of excess syndrome patients and 73.7% of deficiency syndrome patients were correctly discriminated (cutoff value: 0.5).

To identify the variables independently associated with TCM syndromes in CHB patients and to compare the value of SELDI data and clinical parameters in classifying TCM syndromes, logistic regression analysis was performed including SELDI-TOF/MS-measured four significantly different variables displayed in Figure 3 and some clinical parameters listed in Table 1. As shown in Table 2, two protein peaks were independent factors that were associated with TCM syndromes and no clinical parameters were selected. Just as mentioned in Section 3.3, it was proven again that the general clinical data were only good at classifying health and HBC, while the method of SELDI-TOF/MS could be used for TCM syndrome classification. Then peak m/z 4187 and peak m/z 5032 were applied to the classification of different TCM syndrome. And 88% of excess syndrome patients and 73.7% of deficiency syndrome patients were correctly discriminated (cutoff value: 0.5, Figure 4).

### 3.6. Sensitivity and Specificity of Serum Protein Markers for TCM Syndrome Classification

**Figure 5:**
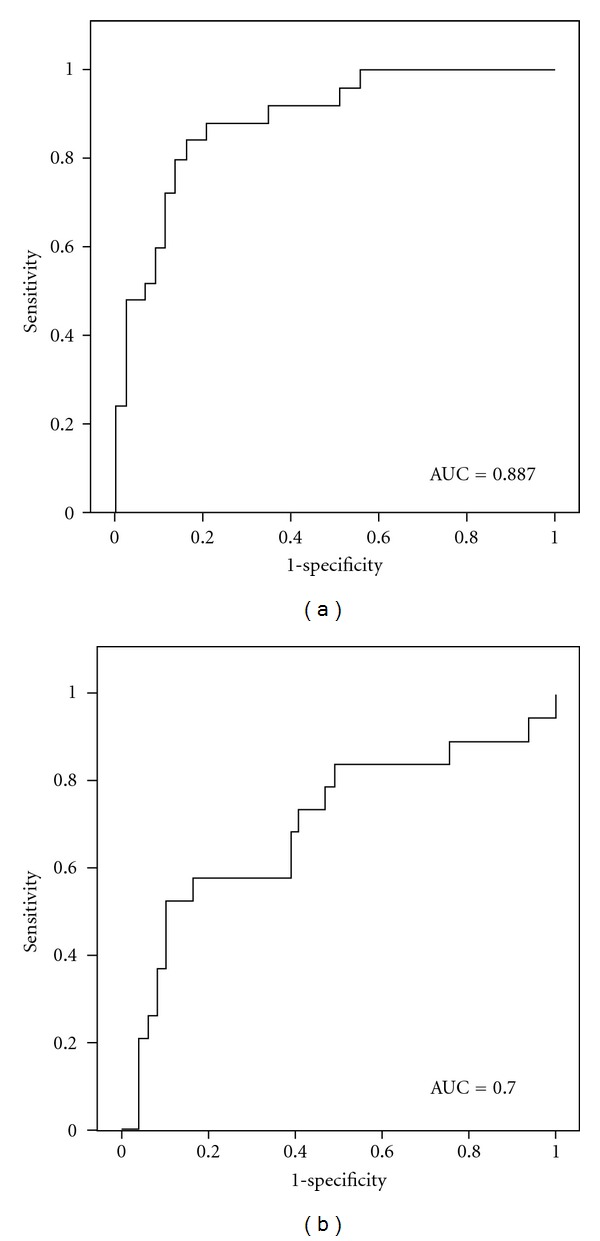
ROC curve for classification of two different TCM syndromes in CHB patients. It was generated combining the peak values of m/z 4187 and m/z 5032. (a) ROC curve for classification of excess syndrome and non-excess syndrome. AUC (area under the curve) = 0.887. (b) ROC curve for classification of deficiency syndrome and nondeficiency syndrome. AUC = 0.700.

To determine the sensitivity and specificity of serum protein potential biomarkers and the usefulness of protein peak quantifications as classification of different TCM syndromes, ROC analysis was conducted. To increase the performance of the classification, the most efficient peak combination was determined using regression analysis. Control group and deficiency syndrome group were put together and defined as the nonexcess syndrome group, so ROC analysis was carried out for discriminating excess syndrome with nonexcess syndrome. The area under the ROC curve for the combination of m/z 4187 and m/z 5032 was 0.887 (Figure 5(a)). In the same way, Control group and excess syndrome group were put together and defined as the nondeficiency syndrome group, and then ROC analysis was performed to discriminate excess syndrome with nonexcess syndrome. The area under the ROC curve was 0.700 (Figure 5(b)). It was suggested that the quantification of these variables by SELDI-TOF/MS was useful to classify excess and deficiency syndromes (Figure 5).

## 4. Discussion

TCM practitioners classify biomedical maladjustments into different syndromes, and each syndrome has its own suitable treatment protocol. Also, considering that the mechanism of disease might not be identical in different people, that is to say, one disease could display several different syndromes, so the same disease may be treated by different therapeutic approaches. The syndrome classification-based individualized therapy is commonly applied in the TCM practice. So we have sufficient reasons to believe that the therapeutic effect will be influenced if excess syndrome and deficiency syndrome of CHB patients were not classified correctly. Therefore, much attention should be paid to the accuracy and the standard of syndrome classification. However, people often argue that the diagnostic approach of TCM practitioners does not meet requirements of objectivity and reproducibility. And TCM diagnosis studies have proved that there exists considerable variability across different practitioners, even when the same patient was diagnosed [21, 22]. So it is essential to find a kind of scientific and persuasive approach for the application of TCM syndrome classification.

 Proteomics is playing an important role in improving our understanding of biologic systems by observing the different interactions among hundreds of proteins simultaneously and aims at studying proteins of human body in the level of integrity. It happens to be in accordance with the viewpoint of TCM, which has always been emphasized on the integrity of human body and the close relationship between human and its environment [3]. In addition, the characteristics of proteomics make it possible to integrate various proteins [23] and easy to study TCM syndrome classification. Comparing with the traditional method that syndromes are classified into groups based on TCM theory and clinical experiences, they can be clustered into specific groups using the approaches of proteomics and bioinformatics. Matsumoto et al. found several proteins for the diagnosis of “Oketsu”, a pathophysiologic concept of Japanese traditional medicine, and differentiated “Oketsu” with “non-Oketsu” successfully [24]. Obviously, it is more scientific and more persuasive. As described in this paper, a proteomics approach was applied, which aimed to provide a kind of accurate and reliable method for TCM syndrome classification.

 In this study, we used the ProteinChip system to analyze and compare the serum protein profiles of excess and deficiency syndromes in CHB patients to define the new potential protein biomarkers for syndrome classification. According to pattern recognition analysis, excess and deficiency syndromes were observed to be clustered into different groups. And four protein peaks were found statistically significant when both groups were compared. On the other hand, syndrome groups could not be classified using general clinical data, and no clinical data were found significantly different between TCM syndrome groups. Among those four possible protein markers, three (m/z 1216, m/z 3168, and m/z 4187) were overexpressed in the deficiency syndrome group and one (m/z 5032) was increased in the group of excess syndrome. Multivariate regression analysis performed by using four significantly different protein peaks from SELDI-TOF/MS data and laboratorial serum markers from clinical data showed the usefulness of two protein peaks (peak m/z 4187 and peak m/z 5032) for excess and deficiency syndromes classification. To observe the sensitivity and specificity of the two proteins, ROC curve analysis was conducted to differentiating excess with nonexcess syndromes and deficiency with nondeficiency syndromes. The area under the ROC curve was 0.887 and 0.700, respectively, suggesting that they could be applied for the classification of TCM syndromes in CHB patients.

 Since one disease could display multiple syndromes in TCM theory, this study focused on several subgroups of CHB patients. It would make protein profiles of different patients keep in the same level of a specific disease and eliminate the interference of diseases for looking for biomarkers classifying different syndromes.

Also, comparing healthy controls with CHB patients of excess syndrome or deficiency syndrome, significant variables were supposed to represent the potential biomarkers about CHB disease and excess syndrome or deficiency syndrome, and the common variables were supposed to represent the potential biomarkers between CHB and healthy group. So in order to find out potential biomarkers for classifying TCM syndromes, those about CHB disease should be eliminated from the significant variables comparing excess syndrome with deficiency syndrome. Therefore, 27 significantly different serum proteins between healthy controls and excess syndrome might be the potential biomarkers for CHB disease and excess syndrome. In the same way, 28 significantly different ones between healthy controls and deficiency syndrome might be the potential biomarkers for CHB disease and deficiency syndrome (Table 3). And 9 common proteins (marked in bold in Table 3) were supposed to represent the potential biomarkers between CHB and healthy group, which should be eliminated from those significantly different proteins between excess syndrome and deficiency syndrome. However, these 9 proteins were totally different with those 4 ones found when comparing between TCM syndrome groups. So it was demonstrated that the interference of diseases to biomarkers had been eliminated.

Most importantly, this study is the first time to classify TCM syndromes in CHB patients by an objective and scientific approach instead of a subjective and experiential one. Our work found the characteristic markers in biochemistry associated with specific TCM syndromes and it will facilitate the development of syndrome classification. Also, it provides an important direction for the understanding and acceptance of TCM theory all around the world. Furthermore, the incorporation of SELDI-based ProteinChip technology into TCM syndrome classification will lead to a new era in the development of TCM to improve treatment efficacy. Our researched results also suggest that TCM syndromes really have their own biological fundament.

## 5. Conclusion

The SELDI-based proteomics found some promising protein profiles and potential biomarkers to classify excess and deficiency syndromes in CHB patients, and it provided an evidence for objective TCM syndrome classification. However, there also exist some limitations in the study, such as the small amount of study population and lack of identification of candidate biomarkers, which would be researched in future study.

## Figures and Tables

**Table 1:** Clinical parameters and TCM syndromes in CHB patients and controls.

Clinical parameters	Excess syndrome (*n* = 25)	Deficiency syndrome (*n* = 19)	Healthy control (*n* = 24)
gender (M/F)	21/4	14/5	15/9
age (year)	38.0 ± 13.4	38.1 ± 11.1	36.4 ± 11.6
BMI (Kg/m^2^)	23.2 ± 3.0	22.1 ± 2.7	21.3 ± 2.1
ALT (U/L)	91.8 ± 116.8	57.4 ± 41.7	20.7 ± 8.7
AST (U/L)	59.8 ± 54.4	50.5 ± 29.1	19.9 ± 5.5
GGT (U/L)	47.8 ± 47.7	56.8 ± 72.0	21.5 ± 9.8
ALP (U/L)	84.2 ± 21.4	90.2 ± 34.7	58.0 ± 20.2
ALB (g/L)	45.2 ± 4.1	44.2 ± 3.5	43.9 ± 5.7
TG (mmol/L)	1.1 ± 0.4	1.4 ± 0.7	0.8 ± 0.3
BA (*μ*mol/L)	10.3 ± 15.9	13.0 ± 18.2	8.0 ± 1.8
TBIL (*μ*mol/L)	19.8 ± 8.3	18.9 ± 5.2	15.0 ± 3.8
PT (s)	13.4 ± 2.0	13.2 ± 1.8	12.7 ± 0.8
HbsAg (+/−)	25/0	19/0	0/24
HBV DNA (+/−)	19/6	11/8	0/24

**Table 2:** Logistic regression analysis for TCM syndrome classification in CHB patients.

Factors	Odds ratio	95% CI	*P* value
m/z 4187	1.349	1.100–1.655	0.004
m/z 5032	0.054	0.005–0.597	0.017
m/z 1216	—	—	0.894
m/z 3168	—	—	0.097
BMI (Kg/m^2^)	—	—	0.301
ALT (U/L)	—	—	0.544
AST (U/L)	—	—	0.452
GGT (U/L)	—	—	0.074
ALP (U/L)	—	—	0.779
ALB (g/L)	—	—	0.093
TG (mmol/L)	—	—	0.262
BA (*μ*mol/L)	—	—	0.206
TBIL (*μ*mol/L)	—	—	0.901
PT (s)	—	—	0.150

**Table 3:** Significantly different peaks between healthy controls and excess or deficiency syndromes.^a^

m/z	Healthy controls	Excess or deficiency syndrome	Change^b^	*P* value
Healthy control versus excess syndrome				
1174	0.20 ± 0.49	0.69 ± 1.17	↑
**2037**	1.71 ± 0.71	3.10 ± 2.24	**↑**
**2269**	0.86 ± 0.38	1.62 ± 1.25	**↑**
2592	0.49 ± 0.27	−0.04 ± 0.34	↓
3203	2.38 ± 1.22	0.13 ± 0.42	↓
3408	1.71 ± 0.80	0.21 ± 0.51	↓
**4104**	32.07 ± 13.66	11.11 ± 6.43	**↓**
4187	11.66 ± 4.61	4.08 ± 3.33	↓
429	7.98 ± 2.88	3.64 ± 2.53	↓
4311	4.79 ± 1.46	1.97 ± 2.70	↓
5032	−0.05 ± 0.33	0.48 ± 0.56	↑
**5497**	1.75 ± 0.79	0.37 ± 0.39	**↓**
**5650**	18.09 ± 6.50	9.10 ± 5.46	**↓**
7027	2.10 ± 0.75	3.33 ± 1.97	↑
7587	1.19 ± 0.45	2.07 ± 1.43	↑
**11732**	0.52 ± 0.26	1.04 ± 0.68	**↑**
14070	0.55 ± 0.23	0.98 ± 0.65	↑
15167	1.95 ± 1.45	4.50 ± 4.42	↑
15354	0.49 ± 0.43	1.22 ± 1.24	↑
**22862**	1.09 ± 0.63	2.31 ± 1.47	**↑**
**23481**	2.25 ± 1.36	4.78 ± 2.65	**↑**
28118	1.72 ± 0.65	2.31 ± 1.02	↑
33516	0.13 ± 0.28	0.37 ± 0.68	↑
38571	0.04 ± 0.02	0.08 ± 0.08	↑
38814	0.04 ± 0.02	0.08 ± 0.08	↑
**46804**	0.03 ± 0.03	0.09 ± 0.08	**↑**
47818	0.02 ± 0.01	0.04 ± 0.04	↑

Healthy control versus deficiency syndrome			
1074	0.49 ± 0.48	0.13 ± 0.37	↓
1210	1.89 ± 1.42	2.64 ± 1.02	↑
1216	6.03 ± 3.50	9.26 ± 3.39	↑
1261	21.14 ± 9.18	29.16 ± 10.80	↑
1440	0.79 ± 1.03	1.36 ± 0.95	↑
2003	1.97 ± 1.04	4.32 ± 2.74	↑
2018	6.92 ± 3.50	13.81 ± 7.86	↑
**2037**	1.71 ± 0.81	3.62 ± 2.51	**↑**
**2269**	0.86 ± 0.47	1.93 ± 1.08	**↑**
3331	3.78 ± 2.69	6.43 ± 3.33	↑
**4104**	32.07 ± 18.72	16.01 ± 11.01	**↓**
5260	0.80 ± 1.44	1.96 ± 1.79	↑
5346	9.05 ± 13.75	21.53 ± 15.00	↑
**5497**	1.75 ± 1.37	0.49 ± 0.50	**↓**
5558	0.97 ± 1.40	1.85 ± 1.32	↑
**5650**	18.09 ± 10.43	10.39 ± 7.67	**↓**
5919	23.56 ± 16.61	40.84 ± 22.76	↑
5947	2.98 ± 3.47	6.79 ± 5.24	↑
6128	4.83 ± 5.95	9.72 ± 6.92	↑
8176	2.82 ± 2.70	4.31 ± 2.73	↑
9723	0.37 ± 0.31	0.83 ± 0.51	↑
10292	1.17 ± 0.98	2.40 ± 1.22	↑
**11732**	0.52 ± 0.36	0.90 ± 0.42	**↑**
15009	0.07 ± 0.09	0.60 ± 1.86	↑
22572	0.30 ± 0.23	0.93 ± 1.18	↑
**22862**	1.09 ± 0.61	2.39 ± 1.35	**↑**
**23481**	2.25 ± 1.53	4.74 ± 2.62	**↑**
**46804**	0.03 ± 0.02	0.08 ± 0.06	**↑**

**^a^**Protein peaks marked in bold were the common biomarkers for CHB disease. ^b^ “↑” and “↓” represent the protein was up- and downregulated in CHB patients compared with the control, respectively.
